# Rad21l1 cohesin subunit is dispensable for spermatogenesis but not oogenesis in zebrafish

**DOI:** 10.1371/journal.pgen.1009127

**Published:** 2021-06-17

**Authors:** Yana P. Blokhina, Michelle A. Frees, An Nguyen, Masuda Sharifi, Daniel B. Chu, Kristi Bispo, Ivan Olaya, Bruce W. Draper, Sean M. Burgess

**Affiliations:** 1 Department of Molecular and Cellular Biology, University of California, Davis, California, United States of America; 2 Integrative Genetics and Genomics Graduate Group, University of California, Davis, California, United States of America; 3 Biochemistry, Molecular, Cellular, and Developmental Biology Graduate Group, University of California, Davis, California, United States of America; Cornell University, UNITED STATES

## Abstract

During meiosis I, ring-shaped cohesin complexes play important roles in aiding the proper segregation of homologous chromosomes. RAD21L is a meiosis-specific vertebrate cohesin that is required for spermatogenesis in mice but is dispensable for oogenesis in young animals. The role of this cohesin in other vertebrate models has not been explored. Here, we tested if the zebrafish homolog Rad21l1 is required for meiotic chromosome dynamics during spermatogenesis and oogenesis. We found that Rad21l1 localizes to unsynapsed chromosome axes. It is also found between the axes of the mature tripartite synaptonemal complex (SC) in both sexes. We knocked out *rad21l1* and found that nearly all *rad21l1*^*-/-*^ mutants develop as fertile males, suggesting that the mutation causes a defect in juvenile oogenesis, since insufficient oocyte production triggers female to male sex reversal in zebrafish. Sex reversal was partially suppressed by mutation of the checkpoint gene *tp53*, suggesting that the *rad21l1* mutation activates Tp53-mediated apoptosis or arrest in females. This response, however, is not linked to a defect in repairing Spo11-induced double-strand breaks since deletion of *spo11* does not suppress the sex reversal phenotype. Compared to *tp53* single mutant controls, *rad21l1*^*-/-*^ *tp53*^*-/-*^ double mutant females produce poor quality eggs that often die or develop into malformed embryos. Overall, these results indicate that the absence of *rad21l1*^*-/-*^ females is due to a checkpoint-mediated response and highlight a role for a meiotic-specific cohesin subunit in oogenesis but not spermatogenesis.

## Author summary

Cohesins are multi-protein, ring-shaped complexes that tether DNA duplexes to one another and are important for nuclear organization of DNA and proper chromosome segregation during cell division. Specialized cohesin subunits play important roles in meiosis, a cell division required to produce gametes. Segregation errors in meiosis can lead to the formation of gametes with the incorrect number of chromosomes (aneuploidy), a major cause of birth defects and pregnancy loss in humans. Here we assess the role of Rad21l1, a cohesin subunit specific to vertebrate animals, by knocking out the gene in zebrafish and examining how oogenesis and spermatogenesis are affected. While males produce normal sperm in the absence of Rad21l1, oocyte production is severely compromised. Oocytes develop partway but then undergo cell death. Deleting *tp53*, a gene involved in sensing cellular stress, prevents cell death and allows oogenesis to be completed, though most of the resulting eggs are severely poor quality. Thus, our work shows that zebrafish oogenesis requires a special cohesin subunit, without which oocytes die. In contrast, spermatogenesis does not require this subunit.

## Introduction

Meiosis is a specialized nuclear division that reduces chromosome ploidy to form haploid gametes. The first division (meiosis I) separates homologous chromosomes and the second (meiosis II) separates sister chromatids. Sister chromatid cohesion is established during the S-phase preceding meiosis I and is required for the molecular events related to pairing and crossing over between homologous chromosomes. Cohesion is also important for holding homologs together in a bivalent structure until meiosis I and sister chromatids together until meiosis II [1–5]. The premature loss of cohesin can lead to gamete aneuploidy, which is a major cause of birth defects or pregnancy loss in women [6,7]. Several lines of evidence point to the premature degradation of cohesin complexes with age [8–13].

Cohesins are multi-subunit ring-like complexes that link two double-stranded DNA (dsDNA) strands together [14–16]. The complexes are composed of two SMC (structural maintenance of chromatin) proteins, which interact to form the ring, and a kleisin subunit that functions to close the ring [17]. Combinations of different SMC and kleisin paralogs carry out a number of cellular functions, one of which is to maintain connections between sister chromatids during meiosis [1,18–21].

A number of proteins that are specific to cohesin complexes that function during meiosis have been identified in multiple organisms [17]. The kleisin subunit REC8 is the best characterized and is conserved in plants, fungi, and animals. Mechanisms that are critical for forming and maintaining the unique chromosome architecture that supports the pairing and crossing over between homologous chromosomes depend on REC8 to form part of the meiosis-specific cohesin complex [1,2,5,18,19,22–30]. Other paralogs of REC8 have been identified in vertebrates, plants, and worms [31]. RAD21L (Rad21l1 in humans and zebrafish) is a kleisin subunit only found in vertebrates [32], though it has been postulated that the *C*. *elegans* protein COH-3/4 may have similar functions [33]. Several studies have linked RAD21L to a role in establishing interactions between homologous chromosomes in mouse spermatocytes [34–39]. The loss of *Rad21l* in mice leads to infertility in males, and age-dependent sterility in females [35]. Although homologs of RAD21L have been identified in other vertebrate genomes, it has not been studied outside of mouse and humans [33].

Zebrafish has emerged as an excellent model to study the chromosome events in meiosis using genetic and cytological approaches [40–43]. Both sexes produce gametes throughout their lives, providing a window to study sexually dimorphic features of female and male meiosis [44,45]. Importantly, hundreds of eggs from individual animals can be analyzed in a single cross providing a quantitative measure of gamete quality. The development of progeny is easily assessed since embryos are transparent and develop outside the body.

Furthermore, lab strains of zebrafish do not have a heterogametic sex with unpaired or partially paired sex chromosomes as seen in many other vertebrate species. This bypasses some of the potentially confounding effects of disrupting meiotic sex chromosome inactivation (MSCI) that can lead to prophase arrest when homolog pairing is compromised [46,47]. For example, the prophase arrest phenotype seen in male *Rad21l* mutant mice has been attributed to the unpaired X-Y sex body [34,35,48,49].

*Rad21l1* is the zebrafish homolog of mouse *Rad21l* and human *RAD21L1*. The aim of this study is to use zebrafish as a model vertebrate organism to study sex-specific roles of Rad21l1. We show that Rad21l1 plays a role in oogenesis yet is dispensable for spermatogenesis. Moreover, deletion of *rad21l1* activates a Tp53-mediated response in females that does not depend on the formation of Spo11-dependent double strand breaks. We propose that Rad21l1 functions at a critical step of oogenesis that may provide insight into errors that lead to increased birth defects and miscarriage.

## Results

**Fig 1:**
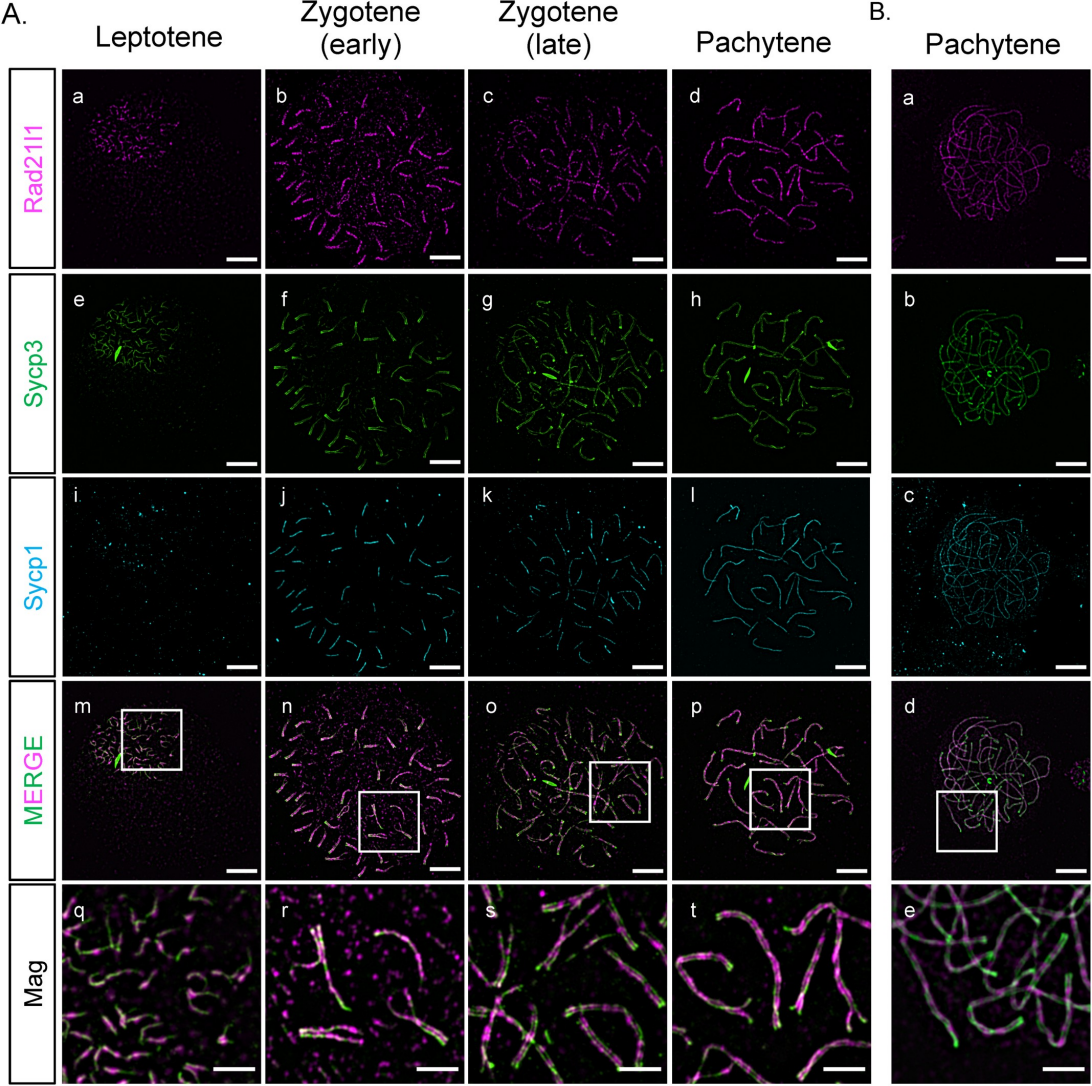
Rad21l1 expression and loading. (A) Rad21l1 loading during prophase I of meiosis in spermatocyte nuclear surface spreads. Rad21l1 (magenta) loads onto chromosome axes simultaneously with Sycp3 (green) and is also dispersed as foci throughout the spread in leptotene. In early zygotene, Sycp1 (cyan) lines start near the telomeres and synapsis extends inward through late zygotene and are present end-to-end along axes. Note: there is some asynapsis in the pachytene nucleus which may indicate that the cell was either in very early or very late pachytene. The merged images are Rad21l1 and Sycp3 channels only. Mag images are magnifications from the Merge panels; the regions magnified are indicated by white boxes. Panel series a-p scale bar = 5 μm. Mag panel series q-t scale bar = 2 μm. (B) Rad21l1 loading during prophase I of meiosis in oocyte nuclear surface spreads. Panels a-e are arranged similarly to the corresponding panels of part (A).

Mouse *Rad21L* and zebrafish *rad21l1* were identified in silico as a kleisin subunit of cohesin by sequence homology to mouse and human *Rad21* and *Rec8* paralogs [32,34]. To determine if zebrafish Rad21l1 protein is a component of axial elements (AE), lateral elements (LE), and/or the transverse filament (TF) of the synaptonemal complex in zebrafish, we created an antibody to the C-terminus region of Rad21l1 (amino acids 329–516) (S1 Fig). Using this antibody, we stained nuclear surface spread spermatocytes and oocytes using immunofluorescence (IF) detection by 3D-structured illumination microscopy (Fig 1). Previously, we and others showed that the AE protein, Sycp3, first loads at subtelomeric regions during leptotene when telomeres are clustered in the bouquet stage. Shortly thereafter, the axes elongate toward the middle of the chromosome during zygotene until they reach full length at early pachytene [40–43]. Synapsis, as detected by IF staining of the transverse filament protein Sycp1, initiates near the chromosome ends and extends inward, slightly trailing the elongation of the AE until pachytene [41,42]. Using the antibody to Rad21l1, we found that foci are dispersed throughout the spread nucleus in leptotene and zygotene yet are concentrated along the unpaired nascent AE (Fig 1A [a-b]). At early zygotene, Rad21l1 continues to load on unpaired AE as they elongate, yet as chromosome regions synapse, Rad21l1 localizes both along and between the axes of the mature tripartite SC (Fig 1A [n-p] [r-t]). By late zygotene and pachytene, the dispersed foci largely disappear and nearly all Rad21l1 protein is found along and between synapsed axes (Figs 1A [c-d] [o,p][s,t] and S2). A similar staining pattern is seen in females (Fig 1B). This localization is similar to that described in a recent study using a different antibody to zebrafish Rad21l1 [43]. Localization to axes and between the axes of the SC is also similar to the localization of RAD21L protein in mice [50]. These results show that zebrafish Rad21l1 is an abundant protein associated with meiotic chromosome architecture starting at leptotene.

### Creating the *rad21l1* mutant

**Fig 2:**
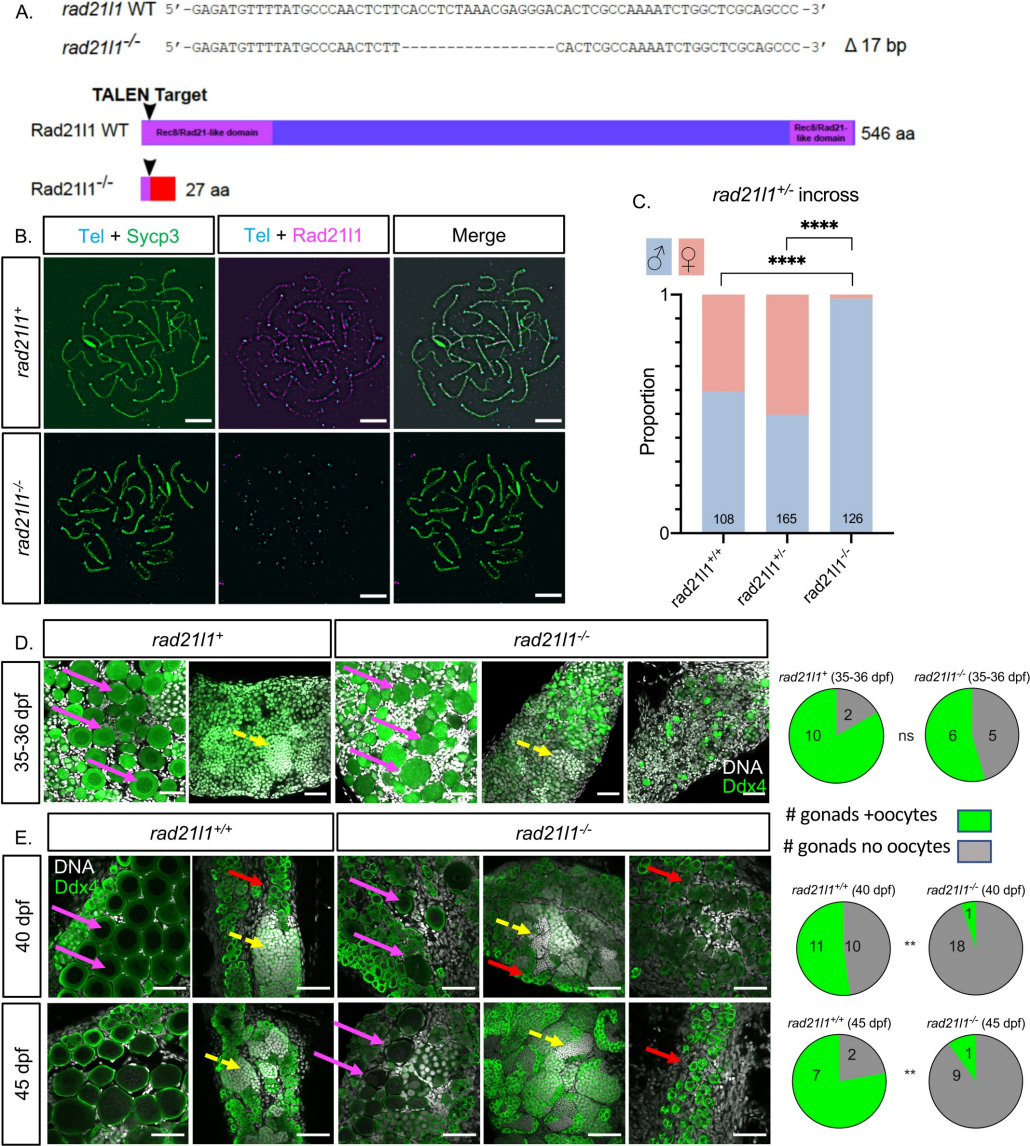
*rad21l1*^*-/-*^ mutants are predominantly male due to late sex reversal. (A) TALEN generated 17-bp deletion leads to a frameshift mutation resulting in a truncated 27 amino acid (aa) Rad21l1 protein with the conserved Rec8/Rad21-like family domains (1–100 aa and 495–543 aa) disrupted or deleted. Rec8/Rad21-like domains (purple boxes); altered amino acid sequence (red box). The ATG translational start site is located at the 4th-6th nucleotide from the end. (B) Spermatocyte nuclear spreads stained for telomeres (cyan), Sycp3 (green), and Rad21l1 (magenta). Rad21l1 forms lines of foci along the Sycp3 axis in *rad21l1*^*+/-*^ spermatocytes. In the *rad21l1* mutant, no lines of Rad21l1 foci are seen. The *rad21l1* mutant spermatocytes can form axes and pair homologs albeit with some asynapsed regions. Scale bar = 5 μm. (C) Sexed offspring of a *rad21l1*^*+/-*^ incross show a depletion of females in *rad21l1*^*-*/-^ fish. Data pooled from multiple crosses. (D) Sections of gonads prepared from 35–36 dpf r*ad21l1*^*+/+*^ *and rad21l1*^*+/-*^ (labelled *rad21l1*^*+*^*)* and *rad21l1*^*-/-*^ fish and stained for DNA (gray) and Ddx4 (also known as Vasa; green). At 35–36 dpf, oocytes are present in 10/12 *rad21l1*^*+/-*^ and 6/11 *rad21l1*^-/-^ samples. Scale bar = 50 μm. Arrows represent different cell types: (pink- diplotene oocytes, yellow- spermatocytes / sperm; red- premeiotic germ cells). (E) Whole mounts of gonads from 40 and 45 dpf are stained for DNA (gray) and Ddx4 (green). At 40 dpf, oocytes are present in 11/21 *rad21l1*^*+/+*^ and 1/19 *rad21l1*^*-/-*^ samples. At 45 dpf, oocytes are present in 7/9 *rad21l1*^*+/+*^ and 1/10 *rad21l1*^*-/-*^ samples. Scale bar = 50 μm. Fisher’s exact test used for all statistical analysis. ns = p>0.05, ** = p<0.01, **** = p<0.0001.

We created a *rad21l1* mutant to assess the meiotic function of this cohesin subunit in zebrafish. The *rad21l1* gene in zebrafish consists of 14 exons encoding a 546-amino acid (aa) protein product (NCBI Reference Sequence: NP_001073519.1). We used TALENs targeted to the second exon to introduce an indel mutation by error prone repair, which we designated as the mutant allele *rad21l1*^*uc89*^. Sequencing of genomic DNA isolated from offspring of founder lines identified a 17 base pair deletion that resulted in a frameshift mutation in the coding region that predicts a truncated protein of 27 aa (Fig 2A). To confirm disruption of Rad21l1 expression, we probed spermatocyte nuclear surface spreads from *rad21l1*^*-/-*^ mutants with the anti-Rad21l1 antibody and found that Rad21l1 was absent (Fig 2B). From this we conclude that *rad21l1*^*uc89*^ is a null allele (hereafter referred to as *rad21l1*^*-/-*^).

### *rad21l1* mutants are predominantly male due to late female to male sex reversal

Sex determination in zebrafish is based on a combination of genetic and environmental factors. While poorly understood, germ cells, and specifically oocytes, are required for female sex determination, the maintenance of the ovary into adulthood, and other external sexually dimorphic features, (for review, see [44,51–53]). If a threshold number of oocytes is produced, the somatic gonad differentiates as an ovary and oogenesis continues. Absent a threshold, the oocytes that were produced undergo apoptosis as the gonad initiates testis differentiation (S3 Fig [54–55]). We therefore assessed the sex ratios of adult progeny from a *rad21l1*^*+/-*^ heterozygous incross based on body shape, color, and morphology of the genital papilla [44]. Among the *rad21l1*^*-/-*^ homozygous mutants, only 1.6% were female (n = 126), while wild-type *rad21l1*^*+/+*^ (n = 108) and heterozygous *rad21l1*^*+/-*^ (n = 165) fish showed sex ratios within the normal range (40.7% and 50.3% females, respectively: Fig 2C). The skew in the sex ratio from female to male suggested that the *rad21l1*^*uc89*^ mutation reduces the overall number of oocytes and promotes sex reversal [41,51].

All zebrafish go through a transient female-like phase during early larval life (approximately 13–24 dpf) and produce Stage IA oocytes that have not yet reached diplotene (pre-follicle stage). In females, oocytes develop further to Stage IB (follicle stage) when chromosomes decondense to form the characteristic lampbrush chromosomes [56]. In zebrafish that will develop into males, the Stage IA oocytes apoptose and the gonad initiates testis differentiation. To test if *rad21l1*^*-/-*^ mutants undergo sex reversal following sex determination, by evidence of the presence of stage IB oocytes, we analyzed gonads from 11 knockout animals and 12 controls (*+/-* or *+/+*) at 35–36 days post fertilization (dpf) stained with DAPI and antibodies to the germ-cell marker Ddx4 (also known as Vasa). We found that (6/11) mutants had stage IB oocytes (Fig 2D pink arrows), slightly less than the control genotypes (10/12; p = 0.19; Fig 2D). These results suggest that at least half of the animals are developing as females following sex determination. Moreover, the oocytes appear intact and not apoptosing, suggesting that they have progressed through early stages of meiotic prophase I [56]. To determine the time window during which *rad21l1*^*-/-*^ mutants undergo female to male sex reversal we examined gonads of wild-type and *rad21l1*^*-/-*^ animals at 40 and 45 dpf stained with DAPI and Ddx4. At both time points, 40 dpf and 45 dpf, the wild-type samples could be easily identified as either female or male based on the presence (female) or absence (male) of oocytes (Fig 2E). While 11/21 (40 dpf) and 7/9 (45 dpf) gonads from control animals contained oocytes, this was true for only a small fraction of mutant gonads (1/19 and 1/10, respectively), indicating a significant decline in oocyte progression (p<0.01, Fisher’s exact test). Moreover, mutant gonads exhibited a broad distribution of gonad morphologies, ranging from i) having Stage IB oocytes (pink arrows), ii) resembling wild-type males (yellow arrows), and iii) having primarily premeiotic germ cells (red arrows). Together, these data suggest that a portion of the *rad21l1*^*-/-*^ males are the product of female to male sex reversal that occurred after sex determination.

Notably, in the two cases where oocytes were seen in mutant gonads, a subpopulation of cells had lampbrush chromosomes indicative of cells reaching the diplotene stage of meiotic prophase (Fig 2E). The left two panels of WT and mutant at day 45 are shown in S4 Fig with an enhanced DAPI channel highlighting the lampbrush chromosomes. These data, in combination with the data collected from 35–36 dpf mutants, suggests that oogenesis progresses to the diplotene stage of meiosis following sex determination, and these stage IB oocytes are removed from the population shortly thereafter due to defects leading to meiotic arrest.

### Some females escape sex reversal

Over the course of the study we found a low number of *rad21l1*^*-/-*^ females. In each instance (n = 3), the females were crossed to wild-type males and gave normal looking embryos. These data indicate that the sex-reversal phenotype associated with loss of *rad21l1* displays incomplete penetrance and that the rare females are fertile. To better assess if the rare *rad21l1*^*-/-*^ females have normal gonad morphology, we carried out histological analysis on one of these animals. Consistent with the fertility of the mutant females, we found that WT and mutant ovaries contained similarly staged oocytes (S5 Fig).

### *rad21l1*^*-/-*^ mutant males produce healthy offspring

**Fig 3:**
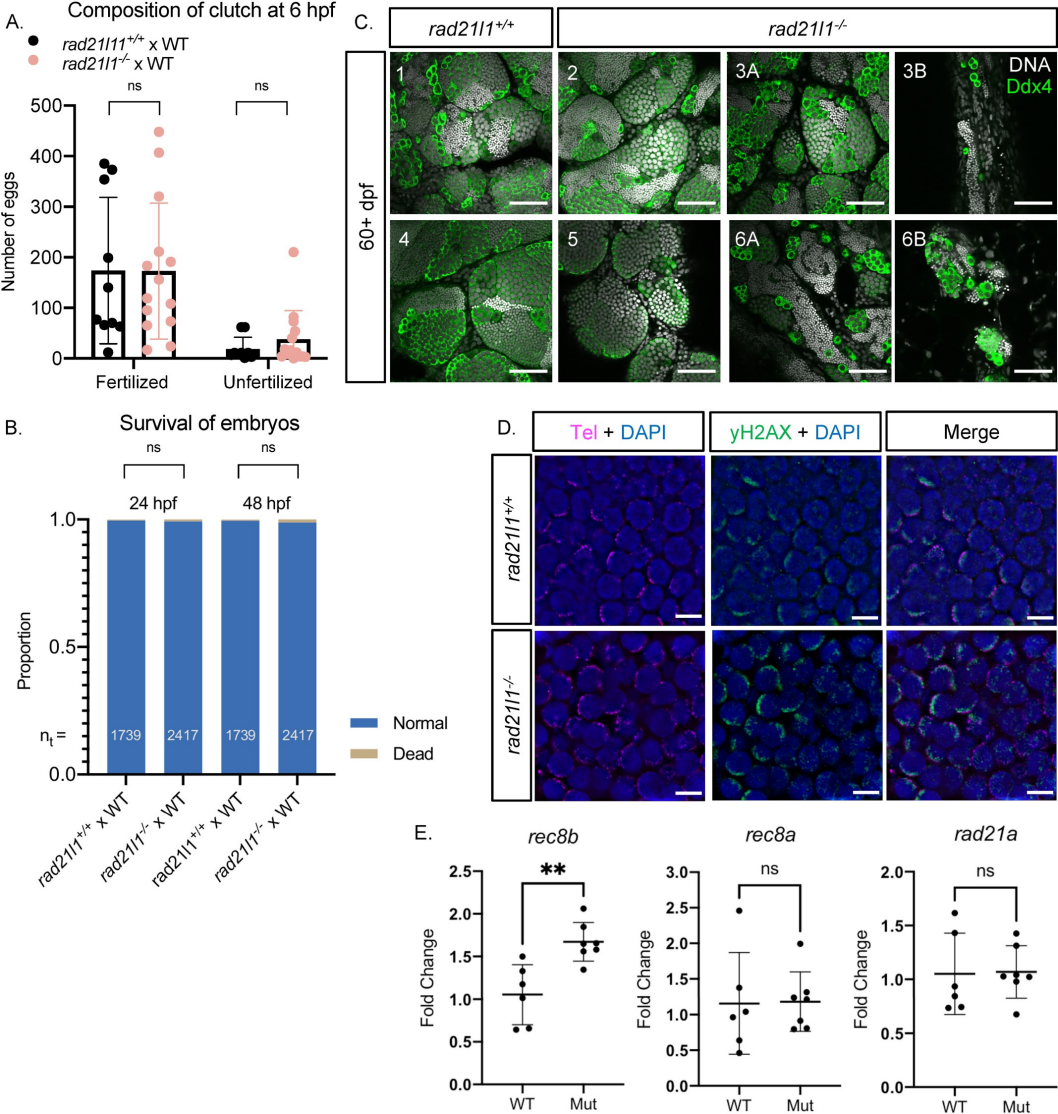
Rad21l1 is dispensable for male fertility. (A/B) Data resulting from test crosses between *rad21l1*^*-/-*^ males and wild-type females to assess fertility and reproductive phenotype. *rad21l1*^*+/+*^ male tank mates were used as controls. No significant difference in the number of eggs the males caused the females to release, the composition of the resulting clutch at 6 hpf, or the survival of the embryos through 48 hpf. Data pooled from 14 crosses over 5 weeks using the same pool of 14 *rad21l1*^*-/-*^ males, 12/14 of which crossed successfully at least once. Unpaired, two-tailed student t-test used for statistical analysis, ns = p>0.05. (C) Whole mount adult testes stained for DNA (gray) and Ddx4 (green) showing a phenotypic range of gonad morphology in *rad21l1*^*-/-*^ males. All samples except #5 displayed large clusters of mature sperm. Images marked as A and B were taken from the same sample to show variation within a single gonad. Wild-type tank mates used as controls. Scale bar = 50 μm. (D) Testes sections stained with a PNA telomere probe (Tel; magenta), an antibody to γH2AX (green), and DAPI (blue), showing that telomere clustering and DSB localization (γH2AX) are normal in the *rad21l1* mutant. Scale bar = 5 μm. (E) Fold change of relative mRNA expression for *rad21l1* paralogs, r*ec8b*, *rec8a*, *and rad21a* in wild-type versus the *rad21l1*^*-/-*^ mutant (Mut). Fold change was determined using the 2^-ΔΔCq^ method. *eF1a* (housekeeping) mRNA levels were used as a reference.

To test if the mutant males are fertile, we set up single pair crosses over the course of several weeks (5 crosses each of wild-type and mutant males per attempt). While 12 of 14 individual mutant males crossed successfully at least one time, 2 males did not cross, even after three attempts. We next used the pool of 12 fertile mutant males to assess if their progeny exhibited developmental defects. The eggs produced by these crosses were collected and categorized at 6 hours post fertilization (hpf) as either fertilized or unfertilized. We found no significant difference in the fertility of the mutant males compared to their wild-type siblings (Fig 3A). All normal, fertilized embryos were further incubated and observed at 24 and 48 hpf. No significant difference in the frequency of survival or the development of progeny of mutant and wild-type siblings was observed (Fig 3B).

### Some *rad21l1*^*-/-*^ mutant males display unusual gonad morphology

Adult *rad21l1*^*-/-*^ mutant males at 60+ dpf had largely normal-appearing gonads as seen by anti-Ddx4 and DAPI staining of whole mounts, however, the exceptions suggest a depletion of early germ cells during development or delayed development of sex reverted males. Of 11 mutant gonads that were stained and imaged, 8 resembled wild-type, two had areas of sparse germ cells, and one contained no germ cells. One possibility is that mutants that reverted to male especially late were unable to recover an appropriate number of spermatogonia during the late development of testes. Notably, even regions that are sparsely populated by germ cells in the mutant adult gonad have sperm, which supports our finding that the majority of mutant males are fertile (Fig 3C).

### *rad21l1*^*-/-*^ mutant males are proficient for forming the bouquet as well as pairing and synapsis of homologous chromosomes

In mice, *Rad21l*^*-/-*^ mutants show defects in telomere attachment to the nuclear envelope [44]. We tested if this was the case in zebrafish by staining mounted gonad sections from wild-type and *rad21l1*^*-/-*^ animals with DAPI to detect DNA, a PNA probe to detect telomeres, and an antibody to γH2AX to detect DSBs. In contrast to what was observed in mice, we found that telomere clustering and DSB localization in mutant sections were indistinguishable from wild-type (Fig 3D). Additionally, by probing Sycp3 localization on nuclear surface spreads, we found that *rad21l1*^*-/-*^ mutant males form 25 paired bivalents, however, asynapsed regions are commonly present (Fig 2B). Together, these data suggest that Rad21l1 is dispensable for meiotic progression and fertility in zebrafish males, though synapsis is often incomplete.

One possible explanation for the ability of *rad21l1* mutants to produce fertile males could be due to genetic compensation by upregulating the expression of a *rad21l1*-related gene, an outcome that is not uncommon among zebrafish knockout mutants [57,58]. Zebrafish have four genes that encode four Rad21l1-related proteins: Rec8a; Rec8b; Rad21a; and Rad21b. We tested if mRNA production of these genes was increased in the *rad21l1*^*-/-*^ mutant males compared to their wild-type controls using RT-qPCR. While *rad21b* was below the level of detection, we observed a 1.6 fold increase in *rec8b* expression in mutants (n = 7) relative to wild-type (n = 6; p = 0.0056; Welch’s t-test) and no significant increase in the expression of the *rec8a* and *rad21a* (Fig 3E). These data suggest that genetic compensation in mutant males, albeit modest, could account for their lack of phenotype. In mice, REC8 and RAD21L proteins localize to chromosomes at the same time in males in a mutually exclusive alternating pattern [36], and show some degree of functional redundancy [37,59]. It should be noted that our RT-qPCR analysis was not possible in mutant females since the females that escape sex reversal appear at a low frequency. It is possible that there could be some genetic compensation in females that allows transit through early stages of meiotic prophase up to diplotene when oocytes are then depleted.

### The *rad21l1*^*-/-*^*;tp53*^*-/-*^ double mutant partially rescues the sex reversal

**Fig 4:**
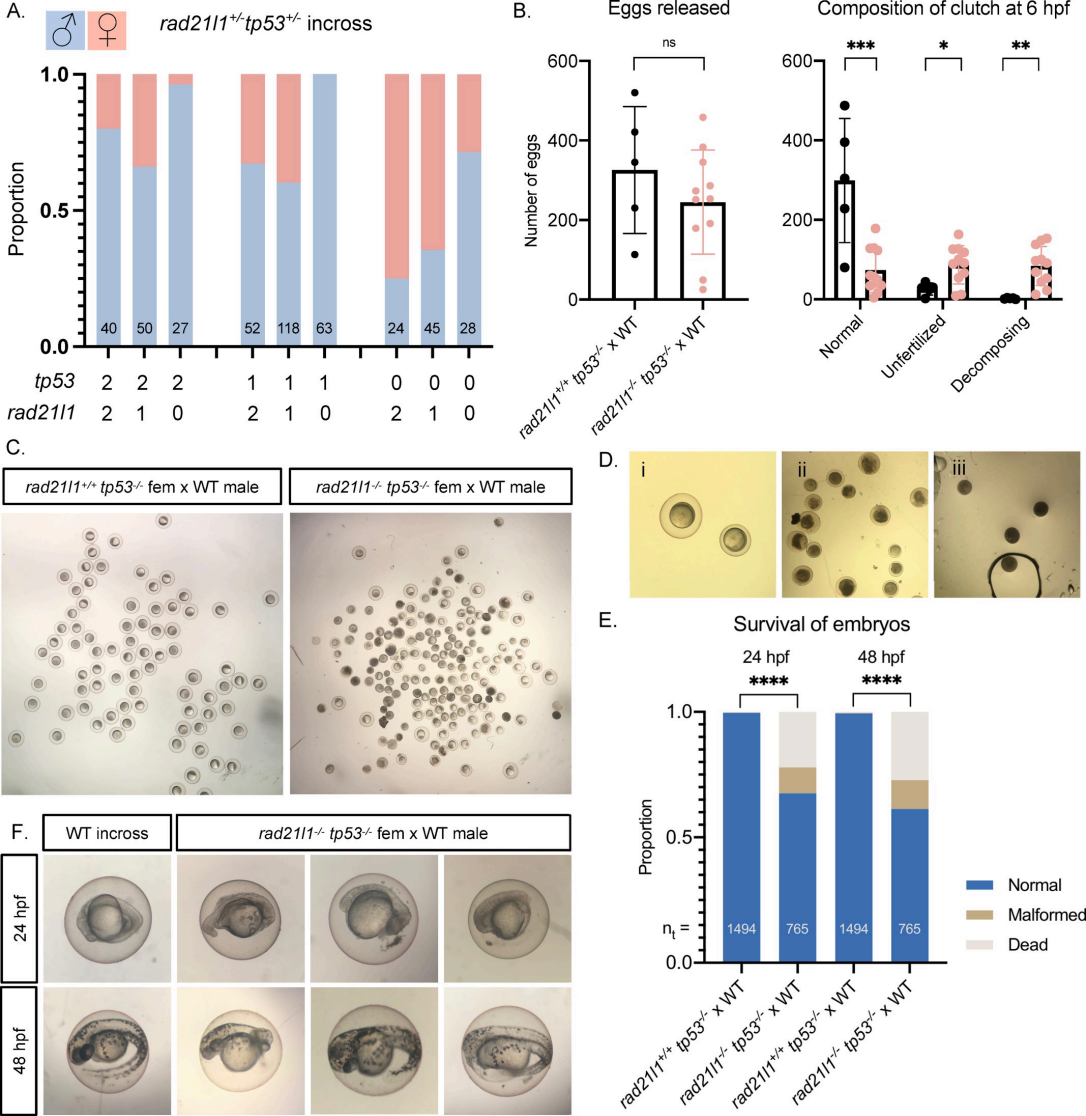
*tp53* knockout restores females to *rad21l1* mutant population, but *rad21l1;tp53* double mutant females produce poor quality eggs and malformed embryos. (A) Sex ratios of all genotypes resulting from a *rad21l1*^*+/-*^;*tp53*^*+/-*^ incross. Data pooled from 3 crosses. 0, 1, and 2 on the x-axis refer to the number of wild-type copies of *tp53* and *rad21l1*. (B) Data resulting from test crosses between *rad21l1*^*-/-*^;*tp53*^*-/-*^ females and wild-type males to assess fertility and reproductive phenotype. *rad21l1*^*+/+*^;*tp53*^*-/-*^ female tank mates used as controls. No significant difference in the number of eggs the females released. *rad21l1*^*-/-*^;*tp53*^*-/-*^ double mutant females release a significantly greater percentage of eggs that fail to be fertilized or display premature decomposition. (C) Representative images of clutches from double mutant and control females at 6 hpf showing lower overall quality of eggs released from double mutant females. (D) Images i-iii show examples of eggs described in the text at 6 hpf. Panel i shows a normal egg (left) and a tiny egg (right). Panel ii shows prematurely decomposing eggs and panel iii shows opaque eggs. (E) Of normal embryos at 6 hpf, 32.4% are dead or malformed at 24 hpf and 38.7% by 48 hpf. Unpaired, two-tailed student t-test used for statistical analysis. (F) Representative images showing the range of malformations seen in developing embryos from *rad21l1*^*-/-*^;*tp53*^*-/-*^ females at 24 and 48 hpf. ns = p>0.05, * = p<0.05, ** = p<0.01, *** = p<0.001.

Previous studies analyzing *brca2* and *fancl* mutants demonstrated that the *tp53* mutation rescues the female to male sex reversal phenotype seen in these mutants, possibly by inactivating a DNA damage checkpoint pathway or by increasing the number of oocytes that promotes female development [60,61]. In mouse spermatocytes, TP53/P53 participates in recombination dependent pachytene arrest [62]. To test if the loss of *tp53* could rescue the *rad21l1*^*-/-*^ sex reversal phenotype, we created a *rad21l1*^*-/-*^*; tp53*^*-/-*^ double mutant carrying a loss-of-function *tp53* missense mutation [63]. To isolate this genotype, we incrossed double heterozygous *rad21l1*^*+/-*^*;tp53*^*+/-*^ mutants and sexed the resulting offspring. We found that while *rad21l1*^*-/-*^*;tp53* ^*+/+*^ and *rad21l1*^*-/-*^*;tp53*^*+/-*^ mutants produced only the rare female (1/27 and 0/63, respectively), 29% (8/28) of the *rad21l1*^*-/-*^*;tp53*^*-/-*^ double mutants developed as females (Fig 4A). These results suggest that deleting *rad21l1* disrupts oogenesis by activating a Tp53 dependent checkpoint. It should be noted though that the *tp53* mutation produces a dramatic skew to female production in the presence of one or two wild-type copies of *rad21l1* (*rad21l1^+/-^ and rad21l1^+/+^*), so the increase of oocytes in general could be contributing, in part, to suppression of the *rad21l1* mutant sex reversal phenotype. Further experiments described below suggest both factors could be acting.

### *rad21l1*^*-/-*^*;tp53*^*-/-*^ double mutant females produce large numbers of decomposing eggs and deformed offspring

Tp53-mediated oocyte depletion is a normal process that occurs during sex determination to form male zebrafish [51,63]. If suppression of the sex reversal phenotype conferred by the *tp53*^*-/-*^ mutation was simply due to an overabundance of oocytes that failed to apoptose, we would expect that *rad21l1*^*-/-*^*;tp53*^*-/-*^ females would produce normal progeny, similar to the rare *rad21l1*^*-/-*^ females described above. Alternatively, if the *rad21l1*^*-/-*^ mutation activates a Tp53-dependent checkpoint due to errors at some step specific to oogenesis, we would expect that the double mutant females would produce oocytes with decreased fertility. To distinguish between these two possibilities, we crossed *rad21l1*^*-/-*^*;tp53*^*-/-*^ females to wild-type males and assessed egg and embryo morphology compared to a *tp53*^*-/-*^ single mutant control. The eggs produced by these crosses were collected and categorized at 6 hpf as normal (fertilized), unfertilized, or decomposing. Fertilized eggs are characterized as being nearly transparent, with a risen chorion and dividing cells. Unfertilized eggs are typically transparent without any signs of decomposition at 6 hpf. Any remaining eggs that had already begun to decompose or did not appear to be correctly formed with a risen chorion were categorized as decomposing.

First, we found that the double mutant females produced high numbers of decomposing eggs and had fewer viable embryos at 6 hpf compared to the *tp53*^*-/-*^ control (Fig 4B and 4C). Many of the decomposing eggs from the double mutants had a more “opaque” appearance than regular decomposing eggs and did not have a lifted chorion (Fig 4D). This is reminiscent of a previously described phenotype where opaque eggs appeared to be oocytes that failed to progress past stage IV of oogenesis [64]. In addition, many of the eggs from double mutant females were smaller in size and had smaller chorions than normal (Fig 4D). Despite this smaller size, these eggs were considered normal if they fit the criteria described above for the *rad21l1*^*-/-*^ male crosses. We tracked the normal embryos to 24 hpf and 48 hpf (Fig 4E). While the majority of embryos developed normally, we found a spectrum of abnormalities, ranging from normal appearance, to almost a complete failure to develop, to severe head or tail truncations (Fig 4F). These findings suggest that the fertility of *rad21l1*^*-/-*^*;tp53*^*-/-*^ females is severely reduced compared to *tp53*^*-/-*^ controls, with much of the defect arising from poor gamete quality (~⅔), and to a lesser extent the formation of dead and malformed embryos. Poor gamete quality could arise from chromosome segregation errors leading to aneuploidy.

Interestingly, in an independent set of crosses we recovered one double mutant female out of 7 that exhibited a normal reproductive phenotype. It is possible that this animal would have developed as a female without sex reversal as was seen for the rare single mutant females (Fig 2C).

### Rad21l1 and Spo11 likely function in different pathways to promote oogenesis

**Fig 5:**
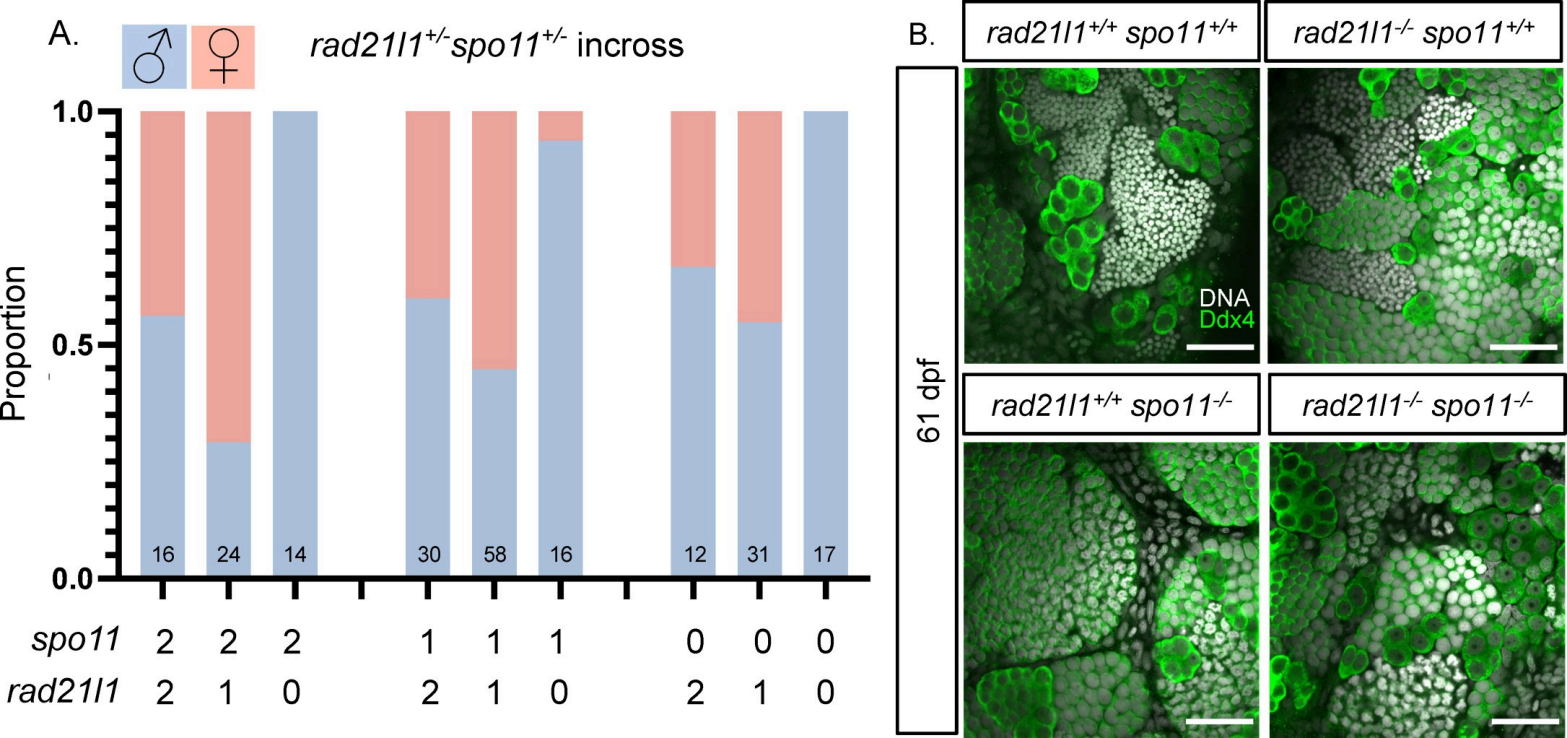
*spo11;rad21l1* double mutants are infertile males. (A) Sex ratios of all genotypes resulting from a *rad21l1*^*+/-;*^*spo11*^*+/-*^ incross. Data pooled from 6 crosses. 0, 1, and 2 on the x-axis refer to the number of wild-type copies of *spo11* and *rad21l1*. (B) Whole mount testes stained for DNA (gray) and Ddx4 (green). WT and *rad21l1*^*-/-;*^*spo11*^*+/+*^ samples display clusters of mature sperm, while *rad21l1*^*+/+*^*;spo11*^*-/-*^ and *rad21l1*^*-/-;*^*spo11*^*-/-*^ samples do not. Scale bar = 30 μm.

Mammalian oocytes respond to defects in processing DSBs by arresting development and undergoing programmed cell death via a TP53/P53 dependent pathway [65,66]. TP53/P53 arrest-inducing mutations in the meiosis-specific DSB repair genes *Dmc1* and *Msh5* are suppressed by the elimination of DSBs by deleting *Spo11* [66]. In zebrafish, *spo11* mutants have normal sex ratios and females are fertile yet give rise to malformed embryos [42,43,67]. We reasoned that if the *rad21l1*^*-/-*^ mutation was inducing meiotic arrest by preventing the repair of Spo11-induced DSBs, eliminating DSBs by deleting Spo11 would rescue the *rad21l1*^*-/-*^ sex reversal phenotype. This was not the case, however, since all of the *rad21l1*^*-/-*^*;spo11*^*-/-*^ double mutants were male (n = 17, Fig 5A). This outcome indicates that Spo11 and Rad21l1 act in separate pathways to promote oogenesis. This is further supported by the non-epistatic phenotype of the *rad21l1*^*-/-*^*;spo11*^*-/-*^ double mutant as seen in Ddx4 stained whole mounts. The *rad21l1*^*-/-*^*;spo11*^*-/-*^ double mutants produce only males as seen in the *rad21l1*^*-/-*^ single mutant yet also fail to produce sperm, as seen in the *spo11*^*-/-*^ single mutants (Fig 5B).

## Discussion

The data shown here support three major conclusions. First, Rad21l1 is an abundant protein associated with meiotic chromosomes and localizes to unpaired axes and along and between the axes of synapsed chromosomes. This pattern is distinct from the localization of either the axis protein Sycp3 or the transverse filament protein Sycp1 of the synaptonemal complex but is similar to what has been seen for RAD21L in mouse [32,34]. Second, spermatogenesis proceeds normally in the absence of Rad21l1, but oogenesis is dramatically affected, as evidenced by female to male sex reversal and reduced fecundity of *rad21l1*^*-/-*^*;tp53*^*-/-*^ double mutant females. We ruled out that this response is due to a failure to form or repair Spo11-induced DSBs. Third, in rare cases, *rad21l1*^*-/-*^ mutant females were recovered that displayed normal reproduction, showing that the sex reversal and poor gamete quality phenotypes have reduced penetrance and variable expressivity. We propose that Rad21l1 plays a role in establishing and/or maintaining cohesin integrity that is important for oogenesis but is dispensable in males and the rare females that escape sex reversal. Thus, the loss of Rad21l1 in zebrafish reveals an increased resiliency of spermatogenesis over oogenesis.

While Rad21l1 protein is present on meiotic chromosomes in both males and females, the majority of mutant males were fertile and produced normal progeny. This is in stark contrast to the phenotype of *Rad21l* mutant mice, where defective synapsis leads to arrest at mid-prophase, resulting in azoospermia [32,34–38,48]. Our finding that the *rec8b* paralog has elevated expression in the *rad21l1*^*-/-*^ mutant suggests that some compensation may be occurring at the mRNA level.

The sexually dimorphic phenotype of the *rad21l1* mutant reveals that fertility in females, but not males, is impacted by the absence of Rad21l11, suggesting some aspect of meiosis specific to oogenesis is compromised. Oogenesis in zebrafish arrests prior to the meiosis I division in the dictyate stage, when individual oocytes in the follicle stage undergo significant expansion before they are released from the ovary [68]. This expansion is coupled to chromatin decondensation to form lampbrush chromosomes [56] and is associated with increased transcription in oocytes [69]. One possibility is that the transition from the condensed to decondensed state is orchestrated by mechanisms that rely on specialized cohesin complexes that include Rad21l1 or Rad21l1 could play a protective role, perhaps by physically shielding DNA or chromatin from potential damage that would trigger a Tp53-dependent checkpoint response. In mice, mutation or decreased expression of cohesin subunits confers increased sensitivity to DNA damage and increased nondisjunction in meiosis I or meiosis II [17,70]. Alternatively, defects arising during DNA replication or early stages of prophase I due to the absence of Rad21l1 may be better tolerated in males than females. At this point however, we cannot rule out that males have a stronger genetic compensation for the loss of Rad21l1 than females. It is also possible that the rare *rad21l*^*-/-*^ females arise due to increased genetic compensation compared to the animals that underwent sex reversal.

Female to male sex reversal is not an uncommon phenotype among zebrafish mutants with defects in early germline development. Mutants that exhibit female to male sex reversal include *fancl* and *brca2*, which are defective for DNA damage repair [43,61,71] and *sycp2*, which is defective for meiotic pairing and recombination [43,61,71]. By contrast, loss of *spo11* or *mlh1* function, which act at different stages of promoting crossovers, does not cause sex reversal, yet females give rise to deformed offspring and the males do not make sperm [42,43,72]. Notably, the *spo11*^*-/-*^*;rad21l1*^*-/-*^ double mutant population was entirely male yet they do not make sperm, indicating that sex reversal in *rad21l1*^*-/-*^ mutants is likely not due to the failure to repair Spo11-mediated DSBs and may instead be mediated by a response to other types of DNA damage [73,74]. In zebrafish, Tp53 has been shown to mediate checkpoint induced arrest in irradiated animals and germ-cell apoptosis in *fancl* and *brca2* mutants [51,63]; [43,61,71].

The presence of oocytes in *rad21l1*^*-/-*^ mutant gonads at 35–36 dpf suggests that sex reversal is occurring after sex determination (S3 Fig). It is possible that the *tp53* mutation suppresses the *rad21l1*^*-/-*^ sex reversal phenotype by simply increasing the number of oocytes to reach a threshold that tips the balance to favor the maintenance of female development [52,53]. Indeed, we found that sex ratios of *tp53*^*-/-*^ females (with one or two wild-type copies of *rad21l1*) were heavily skewed to develop as females compared to genotypes with at least one copy of tp53. However, the poor quality eggs and a high fraction of malformed or dead embryos produced by *rad21l1*^*-/-*^*;tp53*^*-/-*^ females argues that the loss of Rad21l1 sensitizes cells to a genotoxic stress (e.g. errors during DNA replication or key transitions through meiotic prophase) that triggers a Tp53-mediated response leading to oocyte apoptosis during early Stage IB. It will be important to pinpoint the earliest point in oogenesis that is compromised in the *rad21l1*^*-/-*^ mutant through the characterization of oocytes at the pre-follicle stage in 13–20 dpf larval gonads, prior to testis differentiation.

*RAD21L* variants in humans have been linked to increased maternal nondisjunction of chromosome 21 through GWAS analysis [75], and a null mutation in *Rad21l* is linked to age-dependent oocyte depletion in mice [35]. It is important to note, however, young female *Rad21l* null mice are fertile and give rise to normal progeny [35]. While the effect of mutating *Rad21l* in mice is more severe in males, the nature of this arrest is multifaceted. That is, *Rad21l* mutants arrest at pachytene, possibly due to disruption of the process of meiotic sex chromosome inactivation (MSCI) [35,46]. This is consistent with a single-nucleotide polymorphism in human *RAD21L1* linked to azoospermia in Sertoli cell-only syndrome in males [76]. Arrest due to a defect in MSCI would be epistatic to a possible downstream phenotype associated with a *Rad21l* mutation in mice (i.e. arising in diplotene cells), so determining a later role for *Rad21l* during spermatogenesis in mice remains elusive.

Zebrafish, which lacks heterogametic sex chromosomes [77], is an excellent model to directly compare sexually dimorphic phenotypes associated with mutations in meiotic genes. Since continued oogenesis is required to maintain the female ovary, female to male sex reversal provides a unique lens to study defects in oogenesis. Our work shows that the zebrafish homolog of mouse RAD21L and Human RAD21L1 plays an important role in oogenesis.

## Materials and methods

### Ethics statement

The UC Davis Institutional Animal Care and Use Committee (IACUC) has approved of this work under the protocol #20199; For noninvasive procedures (e.g. fin clips for genotyping), zebrafish were anesthetized using tricaine. Invasive surgical methods were performed on fish euthanized by submerging fish in ice water.

### Zebrafish strains

Zebrafish husbandry was performed as previously described [78]. The wild-type NHGRI strain was used in the production of the *rad21l1*^*uc89*^ mutants. Fish used in experiments were outcrossed to the AB strain background 3–4 times. The *spo11*^*-/-*^ strain is in the AB background and described in [42]. The *tp53*^*-/-*^ mutant is described in [63]. All test crosses were performed with wild-type AB strain fish.

### *rad21l1*^*-/-*^ mutant generation

The *rad21l1*^*uc89*^ mutants were generated using transcription activator-like effector nucleases (TALENs) to target exon 2 and genotyped using high resolution melt analysis (HRMA). TALEN target sequences: NG-NI-NG-NH-HD-HD-HD-NI-NI-HD-NG-HD-NG-NG-HD-NI-HD-HD-half repeat NG and NH-HD-NH-NI-NH-HD-HD-NI-NH-NI-NG-NG-NG-NG-NH-NH-HD-NH-half repeat NI. Injected founder fish were raised to adulthood and outcrossed to wild-type fish. The resulting offspring were screened for mutations in *rad21l1* via HRMA and subsequent sequencing. HRMA primer sequences are: Fwd: 5’-CGCCGAGACATGTTTTATGCCC-3’, Rev: 5’-TCAAACACGTGGGCTTTGGT-3’. HRMA was performed with 20X Eva Green dye (VWR, Radnor, PA, Catalog #89138–982) using a CFX-96 real time PCR machine and Precision Melt Analysis software (BioRad, Hercules, CA). Mutants were backcrossed to either AB or NHGRI strain. The sex reversal phenotype was specific to populations genotyped as *rad21l1*^*-/-*^ indicating that it is unlikely due to off-target effects. The phenotype correlation remained consistent through 5–6 crosses.

### Genotyping

Mutant identification: Genomic DNA was extracted and samples were analyzed with HRMA [42]. Primers for *Rad21l1* genotyping were the same as described in the *rad21l1* mutant generation. Primers for *Spo11* were Fwd: 5’-TCACAGCCAGGATGTTTTGA -3’ and Rev: 5’-CACCTGACATTGCAGCA-3’ with an annealing temperature of 61° C. Primers for Tp53 were Fwd: 5’-CTCCTGAGTCTCCAGAGTGATGA-3’ and Rev: 5’-ACTACATGTGCAATAGCAGCTGC-3’. Genomic DNA was extracted and samples were analyzed as described for *rad21l1* mutants except that the reaction was done in 2 mM MgCl_2_ with an annealing temperature of 65° C. Two HRMA runs were required to confirm the three genotypes resulting from a *tp53*^*+/-*^ incross; the first run distinguished heterozygous from homozygous (wild-type and mutant) samples. Homozygous samples were run again under the same conditions but spiked with wild-type DNA to differentiate wild-type and mutant samples.

### Antibody generation

Guinea pig anti-zebrafish Rad21l1 polyclonal antibody production: An N-terminal fragment of Rad21l1 cDNA was amplified with Phusion DNA polymerase (Thermo Fisher Scientific, Catalog #: M0530L) using the following primers: Fwd: 5’-aactttaagaaggagatataccatgTCAAGCTTTTGCCTTCCTGT-3’ and Rev: 5’-tctcagtggtggtggtggtggtgctcAAGCATGCAGAAAAATAAGGCT-3’. The Rad21l1 PCR product was then cloned into pET28b using NEBuilder HiFi DNA Assembly Master Mix (NEB, Catalog #: E5520S). BL21 (DE3) cells containing pRARE and Rad21l1 overexpression construct were grown in 2.6 L of LB with kanamycin and chloramphenicol until an OD600 = 1 and induced with a final concentration of 1 mM IPTG at room temperature for six hours. The Rad21l1 peptide was purified under denaturing conditions using Novagen NiNTA purification resins (Sigma, Catalog #: 70666) according to the manufacturer’s instructions. The Rad21l1 peptide was concentrated to a final concentration of 1 mg/ml in PBS using a 10 kDa centrifugal filter (Sigma, Catalog # UFC901008). The Rad21l1-derived peptide was injected into three guinea pigs by Pocono Rabbit Farm and Laboratory following the 91-day polyclonal antibody production protocol.

### Chromosome spreads and staining

All chromosome spreads and staining were performed as previously described [42,79]. Antibodies and dilutions described in S1 Table.

### Adult testis section and whole mount preparation and staining

Protocols including “whole mount testes staining” and “testes section preparation and staining” were performed as previously described [42]. Antibodies and dilutions are described in S1 Table.

### Whole mount juvenile gonad staining

Juvenile gonad staining was performed similarly to the adult protocol with some modifications:

#### Dissection and fixation

Euthanized fish were decapitated and cut open along the ventral midline to expose the body cavity. Alternatively, an additional cut was made at the anal fin to expose the body cavity if the fish was too small to make a ventral cut. The fish were fixed in 4% PFA in PBT at 4° C for 16–18 hours with gentle rocking. The fish were placed into fresh tubes and washed in 0.2% PBT 3 times for a minimum of 5 minutes each. Gonads were dissected out in PBT and placed into a ceramic 12-well plate, 1 gonad per well.

#### Primary antibody staining

Gonads were washed in an antibody block composed of 5% goat serum and 5% BSA in 0.2% PBT for 1 hour minimum on a 2D rocker at room temperature. Primary antibody chicken anti-Ddx4 [42] was added at 1:500 final dilution. Plate was left rocking gently overnight at 4° C.

#### Secondary antibody staining

The gonads were washed 3 times for a minimum of 30 minutes in PBT, then washed in antibody block as described above. Secondary antibody anti-chicken Alexa Fluor 488 was added at 1:300 final dilution. Plate was left rocking gently overnight at 4° C.

#### Glycerol dehydration and mounting

The gonads were washed 2 times for a minimum of 10 minutes each and dehydrated in a series of glycerol (Sigma-Aldrich, Catalog #: G5516-1L) washes for 1 hour minimum each: 30% glycerol with DAPI at 1:5000 dilution in PBT, 50% glycerol with DAPI at 1:5000 dilution in PBT, and 70% glycerol in PBT without DAPI. The gonads were mounted in 70% glycerol without DAPI on slides with vacuum grease applied to the four corners to hold the coverslip in place. Slides were stored at 4° C until imaging.

#### Histology

The mutant and control females were crossed with wild-type males the morning of fixation to reduce the overall numbers of eggs in the ovaries. Animals were euthanized and decapitated posterior to the pectoral fins and cut open the ventral side to the genital pore using scissors and placed in Bouin’s fixative overnight at room temperature with gentle shaking (10ML/fish). H&E staining of paraffin-embedded gonads was done according to [80]. Images were taken at 5X magnification on a Zeiss Axiophot microscope using a Leica DFC500 camera.

### Imaging

Images of chromosome spreads and whole mount gonads stained with Ddx4 (Vasa) and DAPI were collected at the Department of Molecular and Cellular Biology Light Microscopy Imaging Facility at UC Davis. Chromosomes spreads were imaged using the Nikon N-SIM Super-Resolution microscope in 3D-SIM imaging mode with APO TIRF 100X oil lens. The images were collected and reconstructed using the NIS-Elements Imaging Software. Sections and fluorescent whole mounts were imaged using the Olympus FV1000 laser scanning confocal microscope. Images were processed using Fiji ImageJ software. Only linear modifications to brightness and contrast of whole images were applied. Images of eggs and embryos were acquired using a dissecting microscope. All data and related metadata underlying the findings have been deposited and are available at DRYAD (doi: 10.25338/B8V91Q) [81].

### Test crosses

To analyze fertility, individual mutant fish were placed in a divided mating tank overnight with a single AB strain wild-type fish of the opposite sex. The divider was removed soon after onset of light, and any eggs produced were collected with a strainer, rinsed thoroughly with system water, and placed in a petri dish at 30° C. At 6 hours post fertilization (hpf), embryos were transferred to embryo medium (1X E3 media has final concentrations of 5 mM NaCl, 0.17 mM KCl, 0.3 mM CaCl_2_ dihydrate, 0.33 mM MgSO_4_ heptahydrate, 6 μM methylene blue) and categorized. Fertilized eggs were kept at 30° C and monitored at 24 and 48 hpf for morbidity and mortality. All data related to graphs in Figs 2–4, and 5 are reported in S1 File.

### RNA extraction

Testes were dissected as described above and transferred to tubes containing 200 μL Tri Reagent Solution (ThermoFisher, Catalog #AM9738). Individual testes were homogenized with a plastic pestle (USA Scientific, Catalog #1415–5390) and vortexed every 2 minutes over 10 minutes and kept at room temperature. 20 μL of 1-Bromo-3-chloropropane (BCP) was added to the samples then vortexed and incubated at room temperature for 5 min before a 15 minute centrifugation at 14,000 RPM. 80 μL of the top clear layer was transferred to a fresh RNase-free 1.5 mL tube. 0.8 μL of 20 μg/mL glycogen (ThermoFisher, Catalog #R0551) and 80 μL of 100% isopropanol were added to each tube and the samples were placed at -20° C overnight. The tubes were centrifuged at 14,000 RPM for 30 min and the supernatant was removed. The pellets were washed with 300 μL of 75% ethanol and allowed to air dry. RNA pellets were resuspended in 7 μL of nuclease-free water (ThermoFisher, Catalog #4387937). RNA was quantified using a NanoDrop 1000 Spectrometer (ThermoFisher). RNA was treated with DNase I to remove genomic DNA (ThermoFisher, Catalog #18068–015). First-strand cDNA was synthesized using SuperScript First-Strand Synthesis System for RT-PCR (ThermoFisher Catalog #11904–018).

### RT-qPCR

qPCR reactions were prepared with SsoAdvanced Universal SYBR Green Supermix (Bio-Rad, Catalog #1725271) using cDNA and the following primers (final concentration, 0.05 μM): eF1a (Housekeeping) Fwd: 5’-CTACCTACCCTCCTCTTGGTCG-3’, Rev: 5’-CCTTAAGTAGAGTGCCCAGGT-3’; Rad21a Fwd: 5’-TACCTGCATAGTGAGATGTTCTGT-3’, Rev: 5’- ACAGAACAATGGAGGAAAAACAAC-3’; Rad21b Fwd: 5’-TATCCGTGCATGTGCATTTT-3’, Rev: 5’-CCTCTGGCTACATGATTTGC-3’; Rec8a Fwd: 5’-TGGTGAAGCCTATCCCTCCA-3’, Rev: 5’-CTTCTGGCTCTGGTGGTTGT-3’; Rec8b Fwd: 5’-AGATTCCCCCAAGCAAGTTCA-3’, Rev: 5’-ACAAACTGCATTTAAACTGACCTCT-3’. Cq values were determined for each primer pair and normalized to eF1a control. The fold change between mutant and wild-type was analyzed using the 2^-ΔΔCt^ method. Raw Cq values and calculations are in S2 File.

## Supporting information

S1 Fig Alignment of zebrafish Rad21l1, Rec8a, and Rec8b proteins. Alignment of zebrafish Rad21l1 (ENSDARP00000074083), Rec8a (ENSDARP00000116796), and Rec8b (ENSDARP00000091417) using the Snapgene (v 5.1.4.1) Clustal Omega tool. Yellow shading indicates amino acids of Rec8a and Rec8b that match the Rad21l1 references sequences. The consensus sequence threshold was set at > 50%. Amino acids 329–516 (highlighted) were expressed to create the Rad21l1 antibody in Guinea pigs.
(TIF)

S2 Fig Rad21l1 loading during prophase I of meiosis in spermatocyte nuclear surface spreads. The images are blown up images shown in Fig 1 panel t. A. Rad21l1 (magenta); Sycp3 (green). B. Rad21l1 (gray); Sycp1(blue). Scale bar = 5 μm.
(TIF)

S3 Fig Schematic of the stages of oocyte and gonad differentiation during larval stages of development in zebrafish based on [56,44]. The gonad is considered bipotential starting at 5 dpf and by 30 days animals are differentiated as either female or male. Starting around 13 days, Stage IA oocytes appear, representing the leptotene (L) through pachytene (P) stages of meiotic prophase. The transition to form a testis begins ~20 dpf, after the animals have already started producing Stage IA oocytes. During testis transitioning (~20–30 dpf), the Stage IA oocytes undergo apoptosis and spermatogenesis ensues. In animals that go on to become females, the Stage IA oocytes develop further to Stage IB representing cells in diplotene (D). Note that differentiated females continue to form Stage IA oocytes through adulthood and do not rely solely on the pool generated when the gonad is the bipotential phase.
(TIF)

S4 Fig Oocytes in the *rad21l1*^*-/-*^ mutant reach the dictyate stage. Images are blown up from Fig 2E. The DAPI channel is enhanced to show the lampbrush chromosomes at the follicle stage in wild-type and mutant. The magenta arrows point to the lampbrush chromosomes at the diplotene stage.
(TIF)

S5 Fig H & E staining of gonad from a rare *rad21l1*^*-/-*^ female compared to wild-type. Stages of oocytes indicated (IB, II, III). (A) Ovary section from *rad21l1*^*+/+*^ female; (B) Ovary section from *rad21l1*^*-/-*^ female. Scale bar = 500μm.
(TIF)

S1 Table Antibodies used in this study. (DOCX)

S1 File Master data sheet. (XLSX)

S2 File Final_qPCR_MasterSheet. (XLSX)
