# Supplementary figures and images for: Rad21l1 cohesin subunit is dispensable for spermatogenesis but not oogenesis in zebrafish
Source: PLoS Genet. 2021 Jun 17;17(6):e1009127. doi: 10.1371/journal.pgen.1009127 (PMC8291703; doi:10.1371/journal.pgen.1009127)

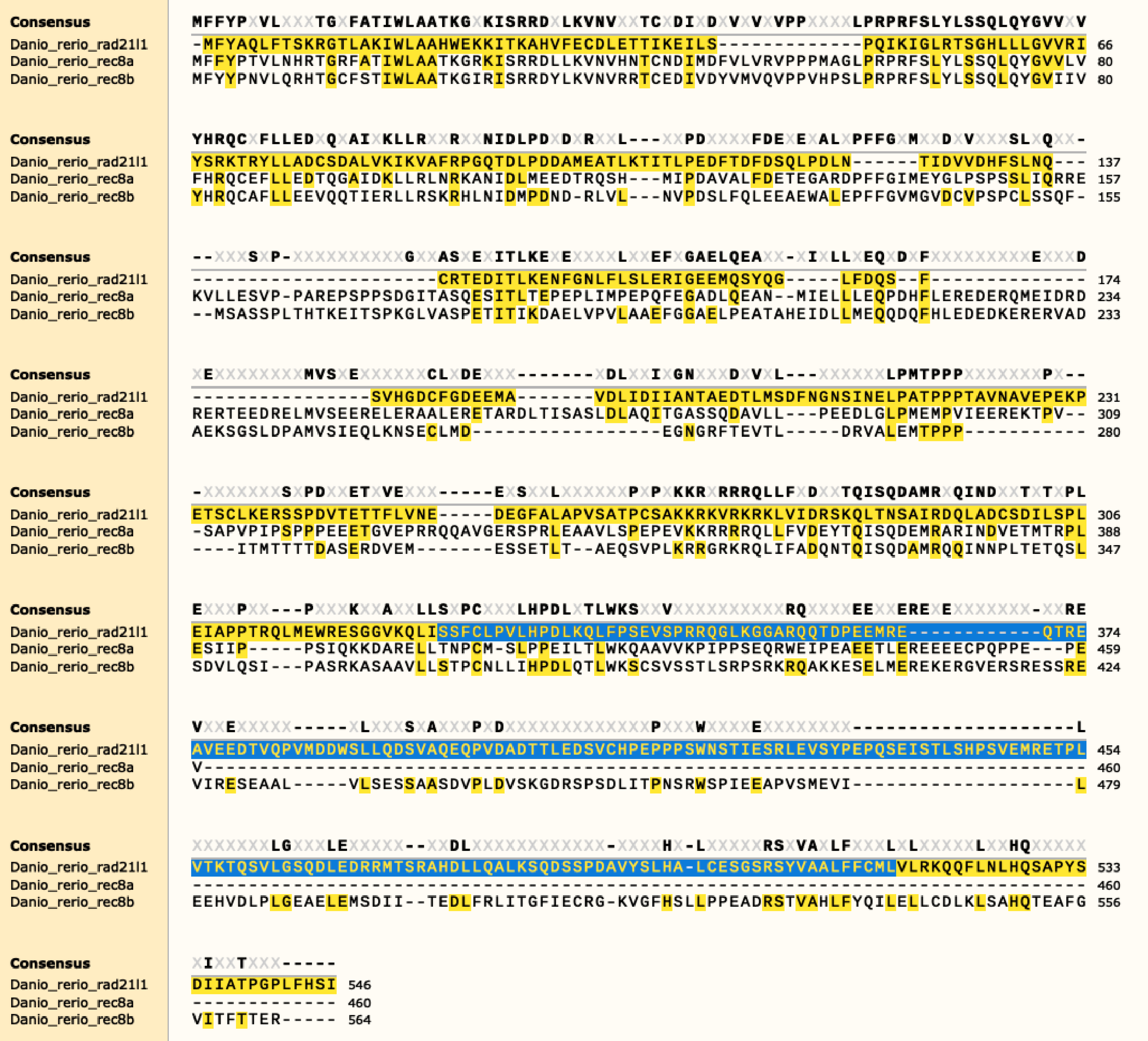

Supplement: S1 Fig — Alignment of zebrafish Rad21l1 (ENSDARP00000074083), Rec8a (ENSDARP00000116796), and Rec8b (ENSDARP00000091417) using the Snapgene (v 5.1.4.1) Clustal Omega tool. Yellow shading indicates amino acids of Rec8a and Rec8b that match the Rad21l1 references sequences. The consensus sequence threshold was set at > 50%. Amino acids 329–516 (highlighted) were expressed to create the Rad21l1 antibody in Guinea pigs. (TIF) [file pgen.1009127.s001.tif]

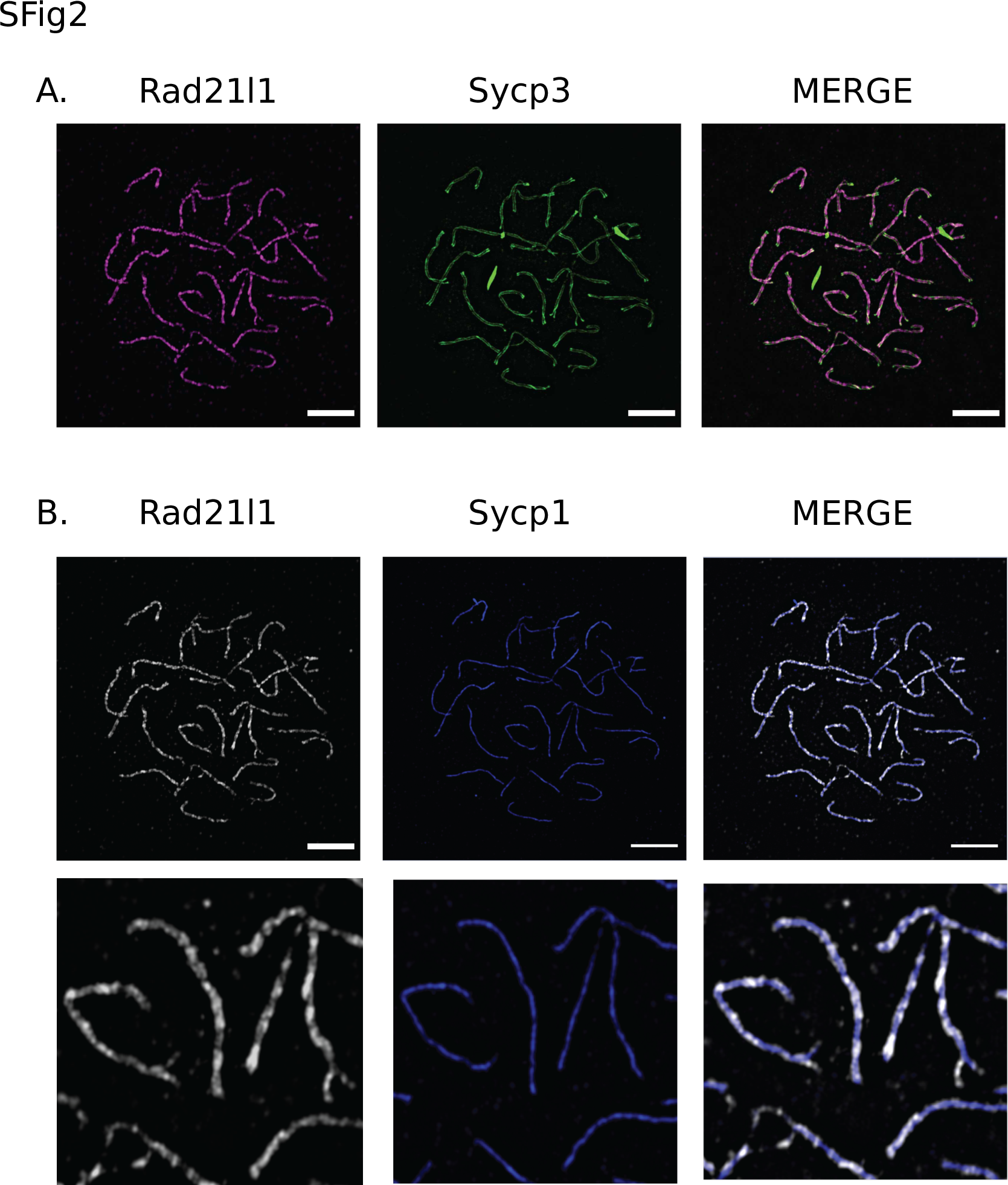

Supplement: S2 Fig — The images are blown up images shown in Fig 1 panel t. A. Rad21l1 (magenta); Sycp3 (green). B. Rad21l1 (gray); Sycp1(blue). Scale bar = 5 μm. (TIF) [file pgen.1009127.s002.tif]

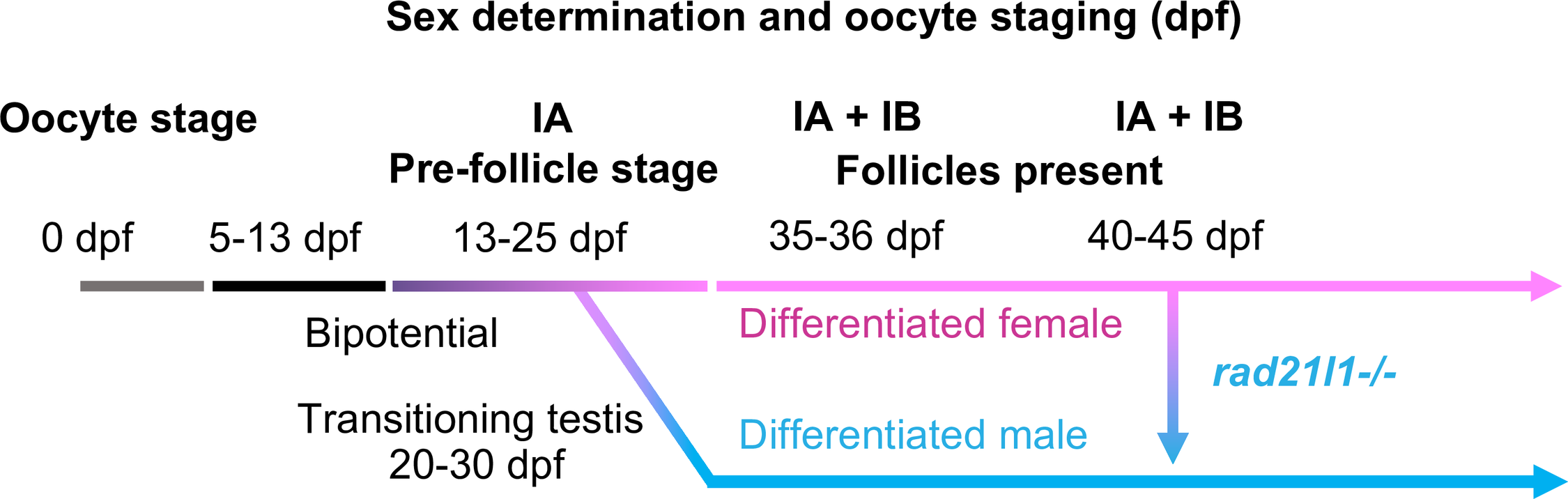

Supplement: S3 Fig — The gonad is considered bipotential starting at 5 dpf and by 30 days animals are differentiated as either female or male. Starting around 13 days, Stage IA oocytes appear, representing the leptotene (L) through pachytene (P) stages of meiotic prophase. The transition to form a testis begins ~20 dpf, after the animals have already started producing Stage IA oocytes. During testis transitioning (~20–30 dpf), the Stage IA oocytes undergo apoptosis and spermatogenesis ensues. In animals that go on to become females, the Stage IA oocytes develop further to Stage IB representing cells in diplotene (D). Note that differentiated females continue to form Stage IA oocytes through adulthood and do not rely solely on the pool generated when the gonad is the bipotential phase. (TIF) [file pgen.1009127.s003.tif]

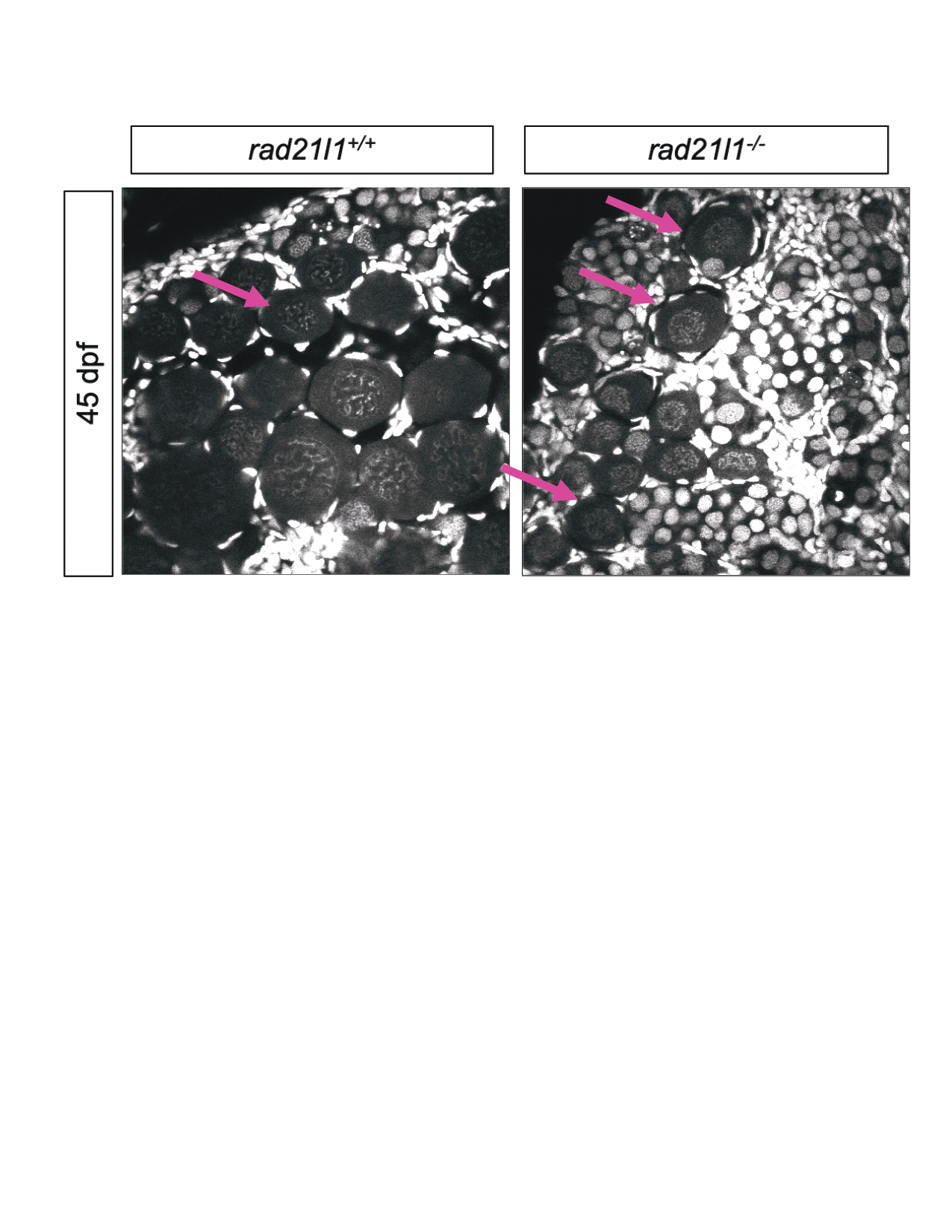

Supplement: S4 Fig — Images are blown up from Fig 2E. The DAPI channel is enhanced to show the lampbrush chromosomes at the follicle stage in wild-type and mutant. The magenta arrows point to the lampbrush chromosomes at the diplotene stage. (TIF) [file pgen.1009127.s004.tif]

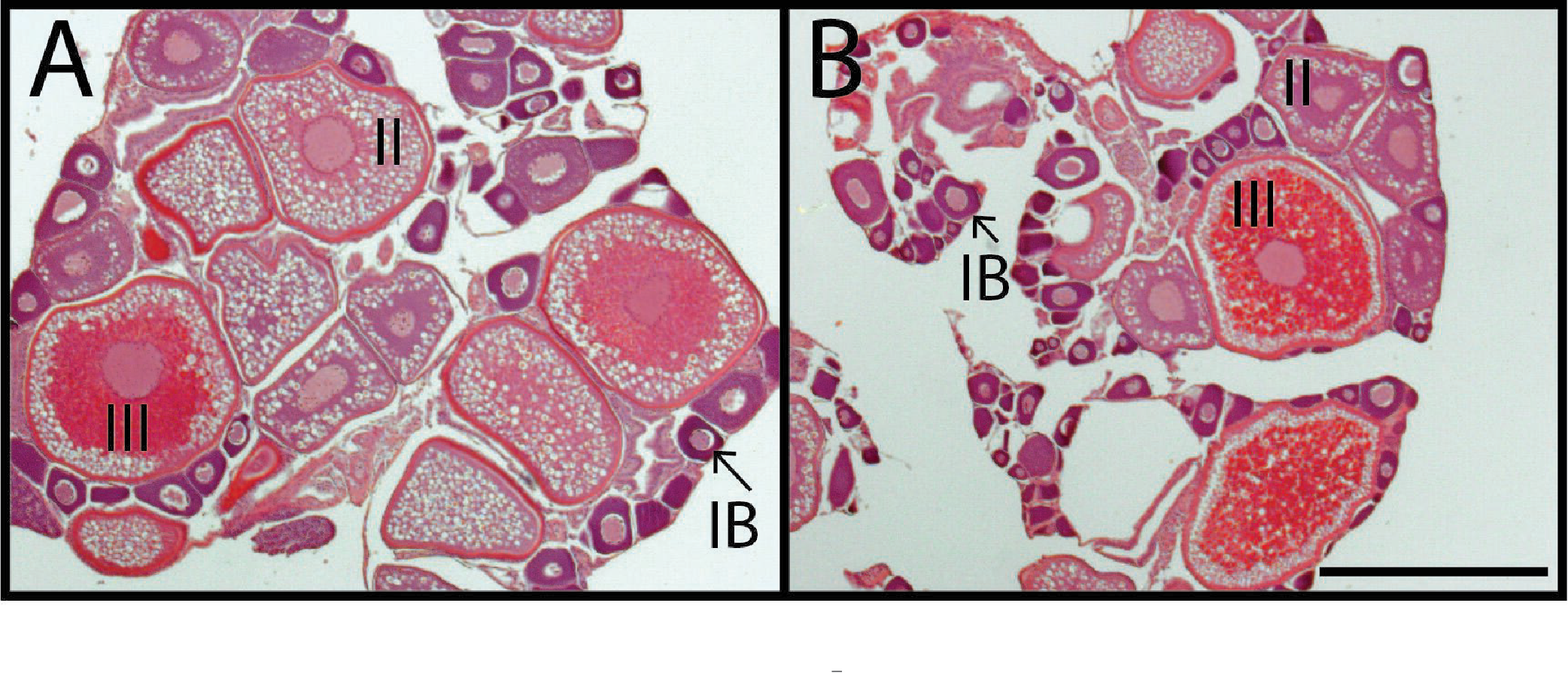

Supplement: S5 Fig — Stages of oocytes indicated (IB, II, III). (A) Ovary section from rad21l1+/+ female; (B) Ovary section from rad21l1-/- female. Scale bar = 500μm. (TIF) [file pgen.1009127.s005.tif]
