# Supplementary material for: Rad21l1 cohesin subunit is dispensable for spermatogenesis but not oogenesis in zebrafish
Source: PLoS Genet. 2021 Jun 17;17(6):e1009127. doi: 10.1371/journal.pgen.1009127 (PMC8291703; doi:10.1371/journal.pgen.1009127)
Supplement: S1 Table — (DOCX) [file pgen.1009127.s006.docx]

**S1 Table. Antibodies used in this study**

| **Name of antibody** | **Company** | **Catalog number** | **Dilution** |
| --- | --- | --- | --- |
| Chicken anti-zebrafish Ddx4 | Burgess lab [1] | N/A | 1:3000 |
| Chicken anti-zebrafish Sycp1 | Burgess lab [1] | N/A | 1:100 |
| Goat anti-chicken IgY Alexa Fluor 488 | ThermoFisher Scientific | A-11039 | 1:300 for whole mounts; 1:1000 for spreads |
| Goat anti-chicken IgY Alexa Fluor 594 | ThermoFisher Scientific | A-11042 | 1:300 for whole mounts; 1:1000 for spreads |
| Goat anti-rabbit IgG Alexa Fluor 488 | ThermoFisher Scientific | A-11008 | 1:300 for whole mounts; 1:1000 for spreads |
| Goat anti-rabbit IgG Alexa Fluor 594 | ThermoFisher Scientific | A-11012 | 1:300 for whole mounts; 1:1000 for spreads |
| Goat anti-guinea pig Alexa Fluor 594 | ThermoFisher Scientific | A-11076 | 1:300 for whole mounts; 1:1000 for spreads |
| Guinea pig anti-zebrafish Rad21l1 | This study | GP2 | 1:200 |
| Rabbit anti-human SCP3 | Abcam | ab150292 | 1:200 |
| Rabbit anti-𝛾H2AX | Gift of James Amatruda | N/A | 1:100 |

1. Blokhina YP, Nguyen AD, Draper BW, Burgess SM. The telomere bouquet is a hub where meiotic double-strand breaks, synapsis, and stable homolog juxtaposition are coordinated in the zebrafish, Danio rerio. PLoS Genet. 2019;15: e1007730.
